# Therapeutic Applications of Magnetotactic Bacteria and Magnetosomes: A Review Emphasizing on the Cancer Treatment

**DOI:** 10.3389/fbioe.2022.789016

**Published:** 2022-04-25

**Authors:** Sai Manogna Kotakadi, Deva Prasad Raju Borelli, John Sushma Nannepaga

**Affiliations:** ^1^ Department of Biotechnology, Sri Padmavati Mahila Visvavidyalayam, Tirupati, India; ^2^ Department of Physics, Sri Venkateswara University, Tirupati, India

**Keywords:** magnetic field, cancer treatment, biocompatible, therapeutic applications., magnetotactic bacteria, magnetosomes

## Abstract

Magnetotactic bacteria (MTB) are aquatic microorganisms have the ability to biomineralize magnetosomes, which are membrane-enclosed magnetic nanoparticles. Magnetosomes are organized in a chain inside the MTB, allowing them to align with and traverse along the earth’s magnetic field. Magnetosomes have several potential applications for targeted cancer therapy when isolated from the MTB, including magnetic hyperthermia, localized medication delivery, and tumour monitoring. Magnetosomes features and properties for various applications outperform manufactured magnetic nanoparticles in several ways. Similarly, the entire MTB can be regarded as prospective agents for cancer treatment, thanks to their flagella’s ability to self-propel and the magnetosome chain’s ability to guide them. MTBs are conceptualized as nanobiots that can be guided and manipulated by external magnetic fields and are driven to hypoxic areas, such as tumor sites, while retaining the therapeutic and imaging characteristics of isolated magnetosomes. Furthermore, unlike most bacteria now being studied in clinical trials for cancer treatment, MTB are not pathogenic but might be modified to deliver and express certain cytotoxic chemicals. This review will assess the current and prospects of this burgeoning research field and the major obstacles that must be overcome before MTB can be successfully used in clinical treatments.

## Highlights

• Magnetosomes have several potential applications for targeted cancer therapy when isolated from the MTB, including magnetic hyperthermia, localized medication delivery, and tumour monitoring.
• MTBs are conceptualized as nanobiots that can be guided and manipulated by external magnetic fields and are driven to hypoxic areas, such as tumor sites, while retaining the therapeutic and imaging characteristics of isolated magnetosomes.
• This review will assess the current and prospects of this burgeoning research field and the major obstacles that must be overcome before MTB can be successfully used in clinical treatments.

## Introduction

**FIGURE 1:**
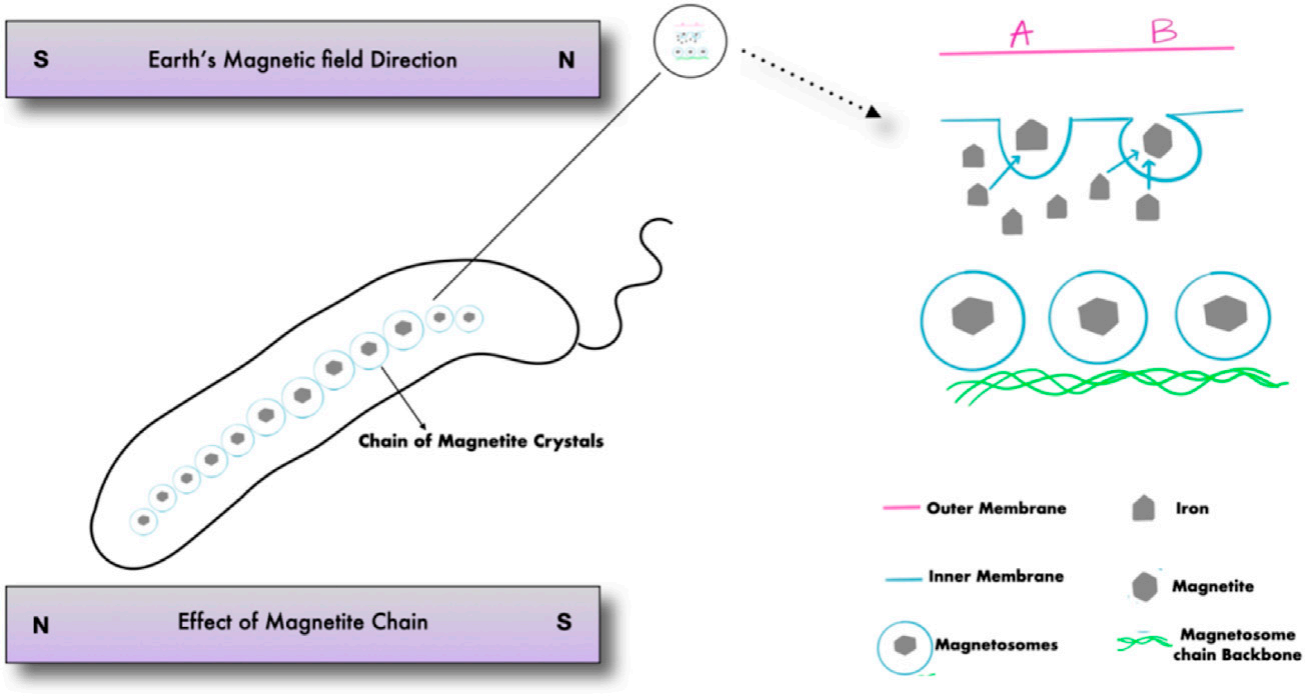
Magnetotactic bacteria and the formation of magnetosomes.

**FIGURE 2:**
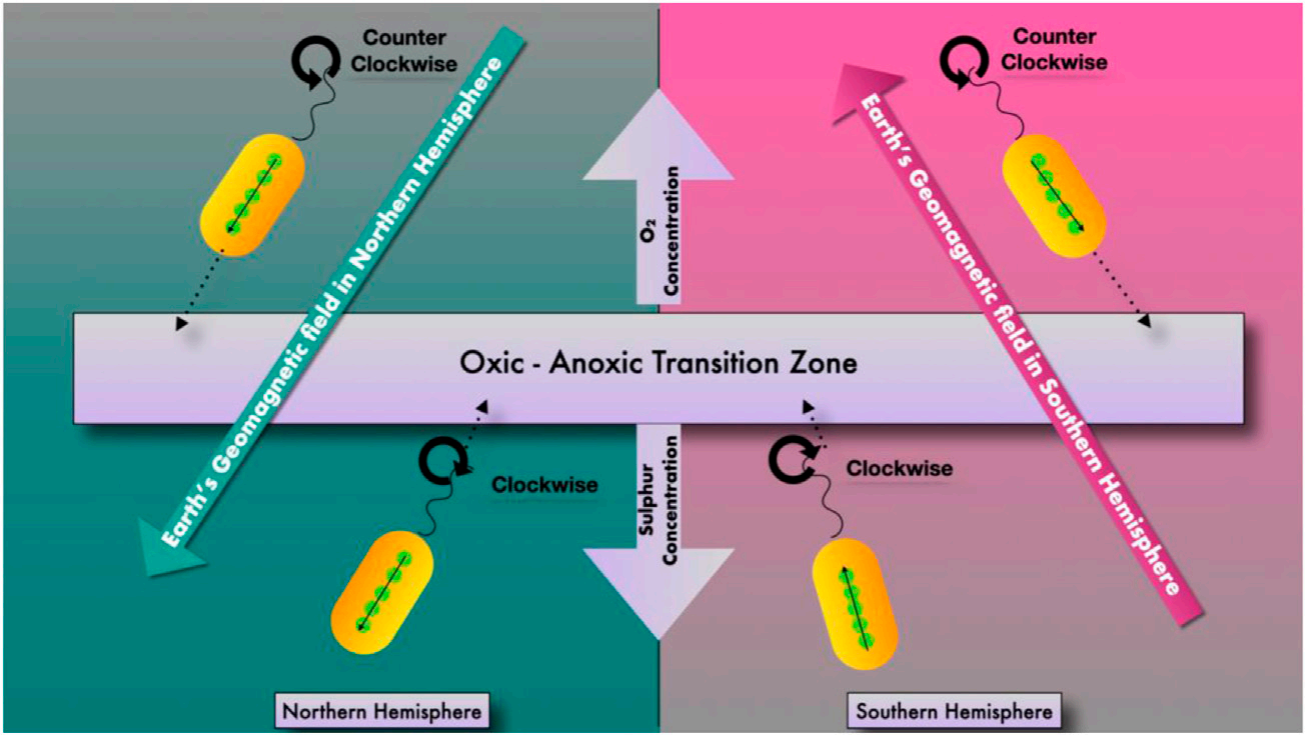
Movement of magnetotactic bacteria in an oxic–anoxic interface.

Magnetotactic bacteria are Gram-negative, motile bacteria that synthesize intracellular crystals of magnetic iron oxide or iron sulfide minerals. Magnetosomes are formed as described in (Figure 1) when mineral crystals and their associated membranes are combined, allowing the bacteria to swim and orient along geomagnetic and external magnetic field lines (Figure 2) (Lefèvre and Bazylinski, 2013). In 1963, Salvatore Bellini was the first to describe these bacteria. Large quantities of bacteria swimming in a single northward direction were initially noticed by Salvatore Bellini, who named them “batteri magnetosensibili, or magnetosensitive bacteria.” Richard P. Blakemore was the first to identify magnetosomes in the cells of magnetotactic bacteria, which he discovered independently in 1974 (Lefèvre and Bazylinski, 2013).

Sediments and chemically stratified water columns include magnetotactic microorganisms. They are more frequent in the habitat’s anoxic zones or at the oxic/anoxic transition zone. In terms of morphology, biology, and phylogeny, they represent a diverse group of microorganisms (Dalan, 2008). Since their discovery, very few individuals of this particular type of bacteria have been identified from axenic cultures, despite multiple study efforts. Even fewer have been well-documented in the scientific literature. Their metabolic adaptability has yet to be discovered (Bazylinski and Frankel, 2004). However, culture-independent methods have been used to examine their diverse morphology and phylogeny. The only genus that has been able to identify magnetotactic bacteria species is Magnetospirillum. These species of the genus have been isolated from a variety of aquatic environments and may now be mass-cultured. They can also be traced genetically. Research into the biochemistry, genetics, and metabolism of magnetotactic bacteria has yielded a wealth of information.

## Habitat

They can be found in all water column types and freshwater, brackish, and marine settings. They were also discovered in damp soils by Dalan and others (Dalan, 2008). They have also been discovered in some damp soils, though it is unclear if they are still around (Bazylinski and Frankel, 2004). Magnetotactic bacteria rely on an oxic–anoxic interface, representing opposite oxygen gradients at the surface and reduced chemicals within sediments or the water column, mainly reduced sulphur species. The most numerous magnetotactic bacteria are found at the oxic-anoxic interface of sediments and in the chemically stratified water column (Zhang et al., 2008). Furthermore, different species of magnetotactic bacteria occupy specific sites at the oxic-anoxic interface, and chemical circumstances are likely to influence these positions. Magnetotactic bacteria biomineralize two magnetic minerals: iron oxide magnetite (Fe_3_O_4_) and iron sulfide greigite (Fe_3_S_4_). While most magnetotactic bacteria create only one mineral, a few can produce both. When the anoxic zones are sulfidic, the magnetite-producing bacteria are frequently found at the oxic-anoxic interface, whereas the greigite producers are found below it. The magnetotactic bacterium, as a result, is an excellent example of a gradient organism (Zhang et al., 2008).

For many years, magnetotactic bacteria have been confined to settings with pH levels near neutral and at room temperature. However, in hot springs, (Lefèvre et al., 2010), described an uncultured, moderately thermophilic magnetotactic bacteria with a possible upper growth limit of 63°C. For optimal growth, these strains have a pH of 9.0. In very acidic settings, they have yet to be discovered (Lefèvre et al., 2010) which is an acid mine drainage.

## Biomineralization and Production of Magnetosomes

Specific characteristics and particular steps in magnetosome biomineralization, on the other hand, remain unknown and may vary depending on the MTB species. Apart from identifying specific genes and chemical precursors, nothing is known about how MTBs manufacture greigite biomineralization. Cells take up extracellular iron ferric or ferrous in the initial step of making magnetite magnetosomes, and it subsequently penetrates past the outer membrane and into the periplasm, where magnetite crystal nucleation in an invagination may be feasible (Komeili, 2012; Rosenberg et al., 2013; Faivre and Godec, 2015). The first known application of MTB, magnetosomes, in this case, was published in 1987. This study used bacterial magnetite magnetosomes isolated from uncultured MTB in a pond (Matsunaga and Kamiya, 1987). However, it was a time-consuming procedure. MTBs were used to immobilize the enzymes glucose-oxidase (and uricase). When immobilized on magnetosomes, these enzymes showed a 40-fold more significant activity than when immobilized on chemically generated magnetite crystals. Later, (Gorby et al., 1988), created magnetotacticum, a technique for extracting magnetosomes from MTB cells. The magnetosomes were purified by magnetic concentration after the cells were lysed in a French pressure cell press. For a variety of reasons, this was a significant development. First, it proved that MTB could be mass-cultured to high cell yields and that many purified magnetosomes could now be studied. It also unlocked the way to the possibility of using purified magnetosomes in specialized applications. The most widely used MTB species in research requiring mass culture and purification of magnetite magnetosomes for application is Magnetospirillum magnetotacticum. In comparison to other MTBs, Ms. gryphiswaldense and MRS-1 strains are both reasonably easy to grow in mass cultures (Gorby et al., 1988). Abiotic magnetite nanoparticles can be produced using a variety of chemical methods, including the sol-gel method (Qi et al., 2011), oxidative precipitation (Li et al., 2010), solvothermal methods (Wang et al., 2010), thermal decomposition (Sun and Zeng, 2002), and microemulsion (Schwab et al., 2004). However, these methods result in magnetite crystals that are inconsistent in size and morphology. Advances in the study of proteins involved in magnetosome formation in MTB led to the development of biomimetic magnetite nanoparticles. These proteins control the nucleation, pH, and redox of the biomineralization process in MTB. They are then employed to make magnetite nanoparticles *in vitro* to fine-tune desired crystal characteristics (Peigneux et al., 2016). Several chemical precursors, including ferrihydrite and high-spin, reduced Fe compounds, have been discovered in magnetosome magnetite biomineralization (Schüler, 2008; Komeili, 2012; Baumgartner et al., 2013; Faivre and Godec, 2015). A mechanism involving disordered, phosphate-rich ferric hydroxide phase transitions into magnetite has also been postulated (Baumgartner et al., 2013). Several greigite magnetosome biomineralization precursors were discovered based on the research of an MTB that makes greigite (Posfai et al., 1998), including mackinawite, which is often tetragonal FeS, and a cubic FeS. Magnetosomes contain unique magnetic mineral crystals with magnetic properties and significant features for their use in various applications. Despite MTB species/strain, these crystals have a stable crystal structure, a small range of crystal size, good chemical purity, and minimal crystallographic defects (Bazylinski et al., 1994; Bazylinski et al., 1995). Although the shape and habit of magnetite magnetosome crystals vary between species, there is a typical pattern of crystals produced by one species of MTB with a specific morphology. The process of magnetite magnetosome biomineralization is tightly controlled genetically and biochemically (Gorby et al., 1988; Amemiya et al., 2007; Komeili, 2012). Mam (magnetosome cell membrane) and mms (magnetic particles membrane specific) genes that encode proteins that play a role in the creation of magnetosome membranes, iron uptake, and the growth and assembly of magnetosomes into chains (Arakaki et al., 2003; Tanaka et al., 2006; Scheffel et al., 2008; Murat et al., 2010; Komeili, 2012). Many of these proteins are now being researched for their possible applications in biotechnology. MamC is one such example, as it is one of the most abundant proteins in the magnetosome membranes of several MTB species (Schüler, 2008; Zhu et al., 2010). It acts as a powerful anchor for a wide range of molecules.

**FIGURE 3:**
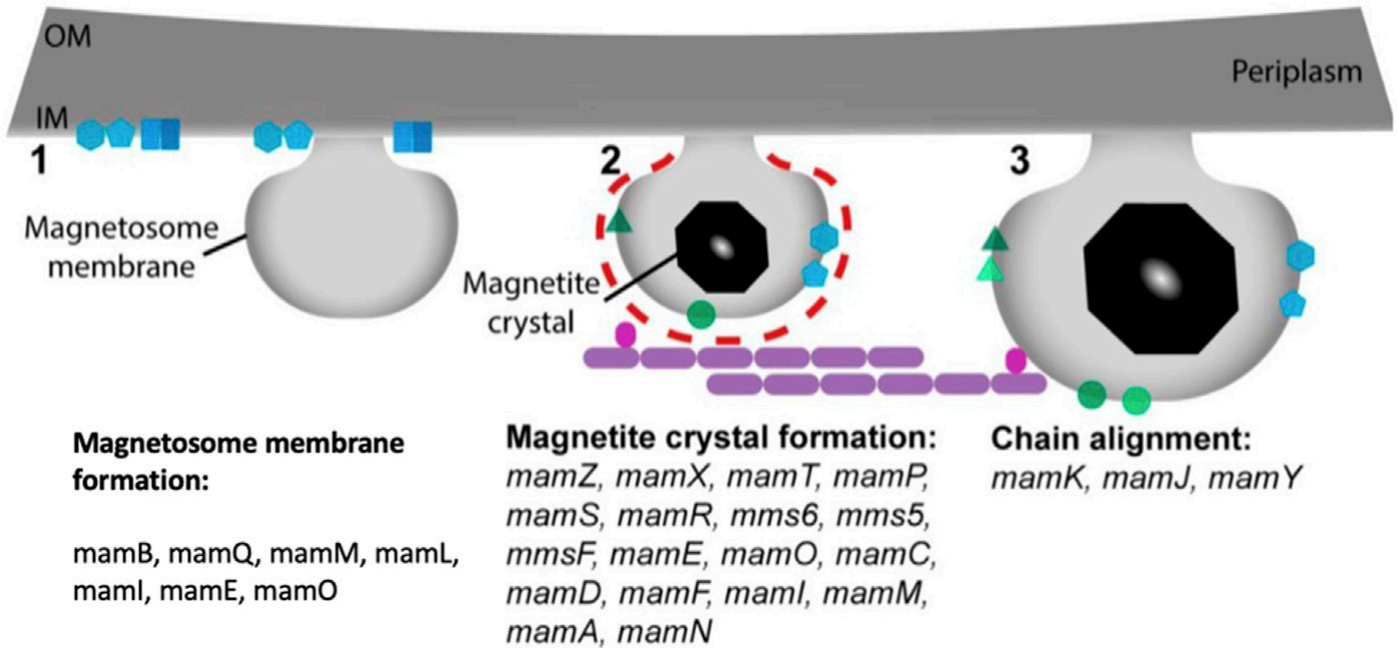
Stepwise process of magnetosomes formation and the proteins involved from membrane invagination.

The magnetite magnetosome biomineralization process necessitates accumulating a substantial amount of iron, which can be absorbed into the cell from the environment in the form of Fe^3+^ or Fe^2+^ There are two types of Fe^3+^ uptake mechanisms: one relies on iron carriers, and another relies on Fe^3+^ reductase to convert Fe^3+^ to Fe^2+^ which is described in (Figure 3) (Chen et al., 2010). MamB, MamM, and MamV proteins are also involved in the absorption and transport of iron. MamB, MamM, and MamV are equivalent in the MS-1 and AMB-1 to MamM, MamB in the MSR-1 (Grunberg et al., 2004). MamB absorbs iron thereby stimulates the generation of magnetosomes and along with MamM, it contributes to direct iron absorption. MamM is hypothesized to promote MamB stability by forming a heterodimer, aiding in iron absorption by MamB (Uebe et al., 2011). MamM, MamN, MamE, and MamO, particularly MamE and MamO, are required for early crystal biological mineralization in MSR-1. MamE has two functions, both of which rely on the development of protease magnetosome crystals and the localization of non-dependent magnetosome crystals (Matsunaga and Okamura, 2003). MamN is related to the hydrogen ion transfer protein, which regulates hydrogen ion efflux in the magnetosome membrane, balancing the potential chemical difference induced by iron ion transport (Yang et al., 2018).

Magnetosomes are made up of magnetic mineral crystals with specific magnetic properties and characteristics useful in various applications. Although the shape and habit of magnetite magnetosome crystals vary per species, there is a basic pattern of crystals that one MTB species creates crystals with a specific morphology (Schüler, 2008; Zhu et al., 2010). Magnetosome-inspired greigite chemically produced nanoparticles show magnetic characteristics comparable to magnetite magnetosome crystals, suggesting that these iron sulfide nanoparticles could also benefit biomedical applications (Feng et al., 2013).

## Therapeutic Applications

### Magnetotactic Bacteria as Anti—Cancer Agents

Bacterial magnetosomes, or MTBs, may aid in diagnosing and monitoring a variety of disorders, including cancer. Bacteria have unique properties that make them ideal candidates for anticancer drugs. Bacteria’s genomes are highly modifiable, and therefore they can be created to circumvent present cancer treatment limits. Radiation and chemotherapy are currently used treatments that damage healthy tissue and do not destroy all cancer cells. Three key aspects can contribute to poor tumor targeting, low tissue penetration, and restricted toxicity to all cancer cells. These issues obstruct efficient therapy, increasing morbidity and death (Sedighi et al., 2019). One of the new research areas in this sector is designing effective therapeutic compound distribution systems (Banerjee et al., 2017; Kutova et al., 2019). One of the main reasons for chemotherapy failure is multidrug resistance (MDR), caused by cancer cells. While several processes aid this, others obstruct the drug’s ability to reach its molecular target. Reduced absorption due to diffusion or endocytosis, receptor involvement, cell membrane protein activity that actively exports substances out of cells, inactivation of drug molecules by xenobiotic detoxification enzymes, and sequestration inside the cell are among these mechanisms (Kartal-Yandim et al., 2016). The nature of cancer cells should be taken into account, and they are unrestricted in their growth and infiltration and can be exploited as therapeutic targets. As a result, many chemotherapeutic chemicals are nonselective and can cause cytotoxicity in healthy cells (Hanahan and Weinberg, 2011). One of the most promising approaches to solving the challenges is to use magnetotactic bacteria and bacterial magnetosomes. Magnetotactic bacteria provide the source of these organelles. According to growing scientific evidence, bacterial magnetosomes could be utilized to treat cancer. The utilization of MTBs and bacterial magnetosomes in biomedical sciences, particularly medicine, is a novel concept. The majority of review articles have described the applicability of these microbes to organelles in broad terms and given a wide variety of solutions and applications. Nanoscale magnetite particles are nanoscale materials with sizes ranging from 1 to 100 nm. According to recent papers in the literature, many of these nanoparticles features depend on their size, which is in the nanoscale (Abdalla et al., 2011). Furthermore, it demonstrates that the coercive force in magnetic materials can be altered by increasing their mechanical strength. It also has an impact on their surface chemistry. This nano-based material has a wide range of environmental applications, including cleaning up contaminated soil and groundwater. Nanoscale magnetic iron compounds are substantially more reactive than typical iron powders due to their tiny particle size. They can also be suspended in a slurry and pumped directly to the affected area. When the elemental metal iron is oxidized in the presence of organic contaminants, the organic contaminants are broken down into simple carbon compounds that are less hazardous. It is also known that oxidizing iron can turn heavy metals into an insoluble form that gets stuck in the soil. The magnetic fluid containing Fe_3_O_4_ nanoparticles in this research was made *via* chemical co-precipitation of ferric and ferrous salts in an alkaline medium utilizing Reimer’s procedure (Abdalla et al., 2011). Protein remodelling influences NP biodistribution, including macrophage capture (Monopoli et al., 2012). Magnetic separation enables the precise extraction of magnetic NPs from complicated biological environments such as blood, fluids, subcellular compartments, and the assessment of the coating which is obtained throughout the particle’s trip *in vivo.* Depending on the stage of cell processing, the coating significantly changes over time (Bertoli et al., 2014). Independent of free biomolecules, the IONP potential of orienting in the magnetic field direction can be employed to precisely investigate the proteins associated with IONPs. Protein adsorption is, in fact, mostly dependent on the surface coating (Lartigue et al., 2012). When IONPs are cultured in a medium containing 10% plasma or pure plasma, which mimics *in vivo* settings, the crown composition varies. This significant finding questions the utility of *in vitro* toxicity testing and highlights the difficulties of simulating NP behaviour *in vivo*. Furthermore, some proteins, such as albumin and apolipoprotein, stabilize particles, whereas others, such as fibrinogen, cause particle aggregation. Surprisingly, populations of differentially enveloped particles coexist in plasma and are handled by immune cells in distinct ways. Macrophages do not capture these populations in the same way or simultaneously as a result. The magnetic behaviour of IONPs is further influenced by the tendency of NPs to aggregate in biological media owing to loss of coating, adsorption of host biomolecules, or active biological processes such as cellular internalization in endocytic compartments (Levy et al., 2011). IONPs have limited freedom to spin and translate in subcellular compartments, and they are subjected to dipole-dipole magnetic interactions. The temperature of transition between superparamagnetic and ferromagnetic behaviour rises as their magnetic susceptibility decreases. As a result, when IONPs are internalized by tumor cells (Di Corato et al., 2014), their heating ability under an alternating magnetic field may be reduced. The potential of particles to heat outside the cells, yet within the tumor stroma and their extracellular distribution (Kolosnjaj-Tabi et al., 2014), makes tumor hyperthermia possible. The sub-compartment confinement of IONPs, however, is beneficial for cell identification by MRI (Smirnov et al., 2006), non-invasive *in vivo* cell migration tracking (Smirnov et al., 2008) and cell manipulation by magnetic forces (Fayol et al., 2013).

MTBs/bacterial magnetosomes are easy to manipulate due to their distinct properties. MTBs, or bacterial magnetosomes, can be employed as anticancer carriers and combined with ligands that target specific cancer cell biological targets. The therapeutic molecule might be delivered entirely within the microenvironment of cancer cells, avoiding healthy tissue. This would allow for focused therapy (Sun et al., 2011; Rosenblum et al., 2018). One of the most significant issues with systemic therapy is low selectivity in chemotherapy. Traditional anticancer medications have the potential to harm both cancer cells and healthy cells. MTBs or bacterial magnetosomes must be safe when exposed to normal cells to be used in cancer therapy. It is critical to establish the pathogenicity and cytotoxicity of MTBs against normal cells in MTB research. Magnetosomes from Magnetospirillum gryphiswaldense were found to not affect the viability and development of murine J774 macrophages in one investigation (Revathy et al., 2017). After being exposed to bacterial magnetosomes, the shape of these cells remained unchanged. Similarly, similar outcomes were seen when bacterial magnetosomes from the same strain were fed to mice fibroblast L-292 cells (Xiang et al., 2007). *In vitro* research on the following cell lines revealed that these bacterial organelles have no minimum toxicity: cervical cancer HeLa (Cypriano et al., 2019), human promyelocytic lung disease HL60, liver cancer H22, and mouse breast cancer line EMT-6 (Sun et al., 2010). After being injected into the bloodstream, BMs were found to remain stable in a rat model. They are not, however, eliminated in the urine or feces (Sun et al., 2009). The liver, on the other hand, has been accumulated. Magnetosomes from bacteria can easily pass the blood-brain barrier (Bai et al., 2013). The conjugation of doxorubicin (DOX, an antibiotic) molecules with BMs, whose protein-lipid membrane is rich in NH_2_ amino groups, is one of the promising ways of modification. These groups are also present in the DOX structure; however, they are not involved in the drug’s activity. DOX and BM conjugates dispersed well in water, where they are homogeneous in shape, and kept their magnetic characteristics. These conjugates were stable in a physiological pH buffer, and considerable drug release was detected in a pH 3.5 buffer. This is a significant characteristic of both successful drug delivery to a malignant tumour and drug particle dispersion within the tumour. As a result, it is reasonable to predict that DOX, when mixed with BMs, will not be released in the lumen of blood vessels Park et al., 2017. The medication associated with BMs had high stability in serum-containing solutions, and its molecules were released slowly under these conditions. According to the cell culture studies, BMX-related DOX retains cytotoxicity to HL60 and EMT6 cell lines *in vitro* (Kuzajewska et al., 2020). The studies of (Qi et al., 2016) yielded similar results in which BMs could pass the blood-eye barrier, resulting in higher survival rates than ARPE-19 cell lines. Alsaiari and others (Alsaiari et al., 2016) have devised an ingenious method for transporting vectors *via* MTBs. The bacteria M. gryphiswaldense was employed as a carrier for ssDNA loaded with gold nanoparticles, which can also be used to control the loading and release of the DNA as a bioimaging agent. Spheroids are three-dimensional *in vitro* cell growth techniques. They are accurate representations of *in vivo* circumstances (Nunes et al., 2019). In the implementation study where AMB-1 magnetotactic bacteria were genetically modified so that the MTB produced magnetosomes linked with RGD peptides targeting agb3 receptors (Matsunaga and Kamiya, 1987). They were bound explicitly to U87 cells and aggregated in GBM tumours after intravenous injection of such magnetosomes into mice. A peptide (P75) that targets EGFR and HER2 was also chemically attached to the magnetosome surface, resulting in selective engagement of these magnetosomes to MDA-MB-468 and SKBR3 cells and higher accumulation in tumours in mice relative to non-target tissues (Edouard, 2020)**.** Spheroids are non-vascularized, early cancers. Their structure, however, is identical to that of cancerous cells. Along their axis, there is a gradient in oxygen, pH, and nutrients. The interior necrotic cells are characterized by hypoxia and a low pH. *In vivo*, these cells are resistant to chemotherapy, and (Afkhami et al., 20112011) described a method for enclosing *Magnetococcus* sp. in enormous monolayer liposomes. This change would improve the efficiency of delivery to target cells. The utilization of MTBs as indirect transporters is a second method for their use. They could be tethered to other carriers and loaded with medication particles. Nanoliposomes coupled with bacteria using the carbodiimide method could be one of these carriers. This provides it an excellent docking location for nanoliposome carboxyl residues. The addition of nanoliposomes and MTBs to bacteria increases biocompatibility (Taherkhani et al., 2014). BM constructions with compounds that differ in structure and interaction with cells have the potential to function as rational co-transporting systems in chemotherapy regimens that use multiple chemotherapeutics at the same time. This could improve the specificity and efficiency of drug transport to the location, so that it lessens chemotherapy’s adverse effects.

### Magnetotactic Bacteria as Nanotheranostics Agents

The research does not indicate how functional MTBs might be exploited as medication transporters. Because of their nature, bacterial magnetosomes are better at this task. Externally induced magnetic field changes can still control MTBs, according to microfluidic system experiments (Martel et al., 2009). This would hypothetically allow these bacteria to go through the human body in specified directions. It would be feasible to control their mobility by altering the magnetic field and bringing them into the environment or tumor. Bacteria are used as specialized nanorobots in this highly beneficial concept (Lu and Martel, 2006; Mathuriya, 2016). In recent decades, much of the research has focused on theranostic nanorobots, which is notably true in the treatment and detection of cancer (Neuberger et al., 2005; Sun et al., 2008). These nanoparticles are simple to make in the lab and offer several exciting characteristics for usage as nanorobots. They are microscopic (5–100 nm) and have the ability to interact with cancer cells. Their biocompatibility (e.g., iron oxide nanoparticles) reduces the likelihood of unpleasant reactions once they are ingested. They can, however, be utilized remotely to modify their magnetic characteristics and cause them to behave in the tumor region (Gao et al., 2009; Leng et al., 2018). This is how magnetic nanoparticles can be used to detect tumors using magnetic radiation imaging contrast agents, magnetic particles imaging, and other methods, as well as to treat and eliminate tumors using drug delivery methods such as magnetic hyperthermia, photothermia, and other methods (Sun et al., 2008; Ortega and Pankhurst, 2013; Plan Sangnier et al., 2018). MTBs and BMs are ideal candidates for targeted cancer therapy from a medical standpoint. However, only a few chemotherapeutics have been studied using MTBs and BMs as drug carriers to date. They were tested on animal models and *in vitro* models using continuous cell lines. As a result, more study is needed to explore a wider spectrum of cell and tissue types, as well as therapeutically used medications. Furthermore, at this point, primary culture analyses and increased research employing spatial models such as spheroids that better depict the interactions occurring in the tumor microenvironment are advised. There is still a lack of data to explain the concerns surrounding the pharmacokinetics and biocompatibility of MB-drug conjugates. Further research should definitively explain whether magnetosomes considerably improve the efficiency of transported chemotherapeutics or whether their role is restricted to a carrier that precisely delivers the active ingredient to cancer cells. It has become clear that utilizing tumor-recognizing biomolecules to accompany MNPs to their targets can improve the efficiency of nanoparticle-based cancer magnetotherapy. Biomolecules such as monoclonal antibodies are commonly employed today. Aptamers, like antibodies, are proving to be effective agents for delivering nanoparticles to specific locations. Aptamers, like monoclonal antibodies, bind to biological targets with remarkable specificity. Several effective aptamer-based magnetic nanoplatforms for targeted cancer therapy have been created to date. As a result, magnetic nanotheranostics, based on diverse ligand and/or drug functionalized nano constructions, provides a new avenue for sophisticated differential diagnostics and metastatic cancer therapy (Belyanina et al., 2017).

Aptamers guide nanoparticles to cancer cells, increasing their accumulation in the tumour and selectively inducing photo- or thermal damage to aberrant cells (Shi et al., 2014). Cancer research has also shown overexpression of folate receptors on the surface of tumour cells; as a result, nanoparticles functionalised with folic acid bind to tumour cells with high affinity (Yu et al., 2012). Acids have also been widely used for the targeted distribution of nanoparticles. Folic acid, for example, is a water-soluble vitamin B6 that aids in rapid cell growth and division, particularly during embryonic development. Carbohydrates have also established themselves as target ligands; for example, the asialoglycoprotein receptor (ASGP-R), which is found primarily in hepatocytes (Kim et al., 2005), readily binds galactose, mannose, and arabinose; as a result, these carbohydrates can be used as agents for targeted delivery to the liver (Yang et al., 2010).

Magnetotactic bacteria produce chains of completely stoichiometric magnetite microcrystals in a variety of species. These nanocrystals are encased in a biocompatible membrane. Magnetotactic bacteria can detect magnetic fields and can be guided, modified, and guided externally. They can also use MRI, magnet hyperthermia, or medication delivery to treat and identify cancer (Behkam and Sitti, 2008; Benoit et al., 2009; Alphandéry et al., 2011a; Felfoul et al., 2016). Magnetosomes are frequently distributed in one or more chains in non-spherical cells (Schüler, 2008). A magnetic crystal is encased in each magnetosome membrane, and it is a lipid bilayer with many proteins in it. The invaginations to the cytoplasmic membrane produce this membrane (Murat et al., 2010). The vesicle formed by membrane pinching may be critical in establishing the chemical/redox environment for magnetite and greigite crystal development and nucleation. It also regulates the size and morphology of the cells (Gorby et al., 1988; Grunberg et al., 2004).

### Magnetotactic Bacteria in Drug Delivery

According to a recent review on the subject, the advantage of MTB or magnetosomes as drug delivery systems is that an applied magnetic field can be employed for drug administration to reach the intended tissue without damaging non-targeted areas. *Magnetococcus marineus* strain MC-1 was employed to deliver drug-loaded micro liposomes into mice’s hypoxic colorectal cancer hypoxic regions. The results demonstrate that when nanocarriers are coupled with MTB, their therapeutic index decreases (Felfoul et al., 2016). In this study they used two strains of mice (A/J and C57BL6), in which no significant changes observed in the production of inflammatory cytokines between PBS and MC-1 injections and these were seen in the systemic response to MC-1 cells. Immunogenicity tests were carried out on two separate strains of mice, using varying dosages of MC-1 and collecting blood and organs at various periods after IV infusion of MC-1 bacteria. In comparison to the liver, spleen, lungs and blood plasma showed the greatest amounts of inflammatory cytokines after IV injection with *P. aeruginosa*. Inflammatory cytokine levels in the liver, spleen, lungs, and plasma were not significantly increased by MC-1 cells. Rats were given 1 × 10^8^ MC-1 cells intravenously, and biochemical and haematological tests were done after 6, 24, and 72 h. Our preliminary findings showed that injecting MC-1 cells did not cause inflammation because no changes in blood counts and biochemical markers were within normal limits. A marine bacterium, Mc. marinus strain MC-1 would not survive in a mammalian body. However, this has not been investigated or evaluated before this investigation. Felfoul and his colleagues (Felfoul et al., 2016) have discovered that when Mc. marinus cells are put into mice, they are clinically safe and have no negative consequences. Because of the overall immunogenicity of Gram-negative bacteria cell walls, this unexpected result was unexpected (Lüderitz et al., 2016). Mc. marinus cells were still alive and motile after being injected into mice in the peritumoral region, which was surprising. These cells were able to penetrate deeper tumors than passive agents such as microspheres or dead Mc. marinus and demonstrated both magnetotactic and aerotactic effects. Mc. Marinus strain MC-1 is a marine bacteria that, while not tested, would not thrive in a mammalian organism. To ensure safety, extensive research into the effects of introducing MTB into living species should be conducted. Magnetosomes tend to be preferentially examined and employed in any biomedical application since their membrane lacks the lipopolysaccharide of the Gram-negative cell wall, which is known to act as an endotoxin (Wang and Quinn, 2010; Lüderitz et al., 2016). These structures will not be able to grow, infect others, or elicit a significant immune response. Martel (Martel, 2017) discusses the potential of drug-loaded MTB as “smart therapeutic agents” for an effective delivery method that targets a specific location or organ in the body.

### Magnetotactic Bacteria in Magnetic Resonance Imaging

**FIGURE 4:**
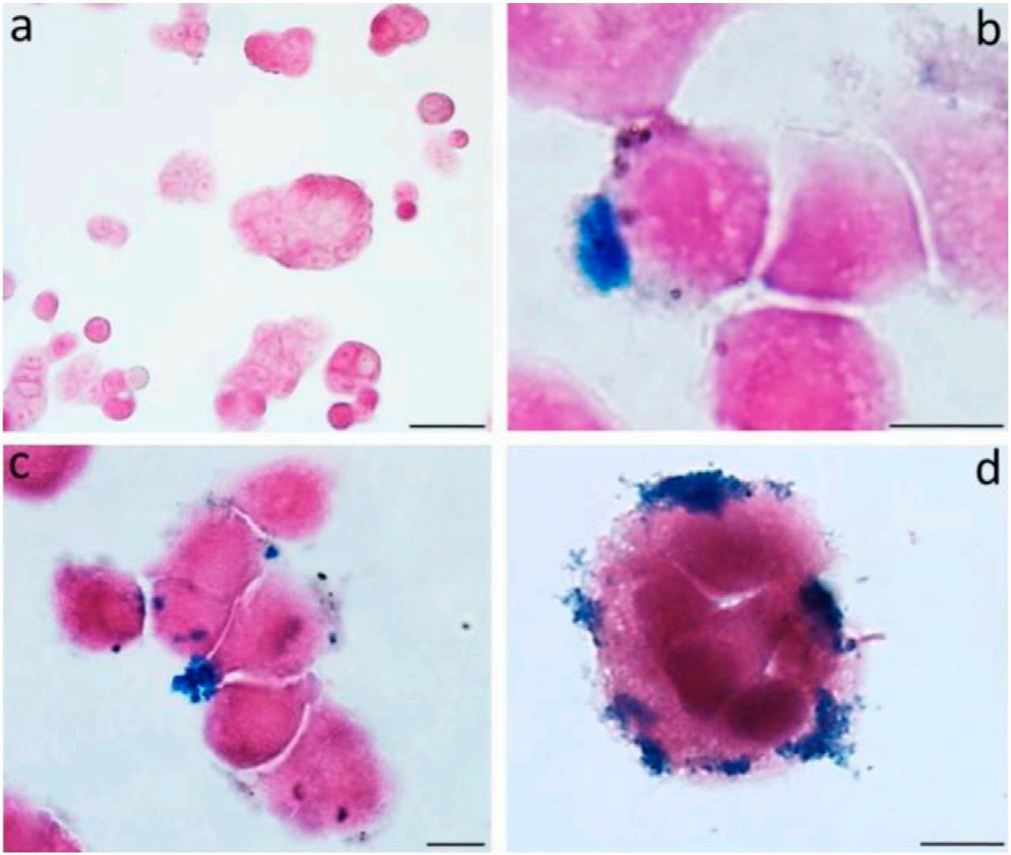
The figure shows the untreated cells **(A)** and MNs-treated cells **(B–D)** observed after 24 h. Prussian blue evidences the iron deposits.

MRI is commonly used to diagnose and evaluate cancers. Magneto spirillum, a bacteria, is used in this radiologic investigation. It is a magnetic microbe with microaerophilic characteristics. It comprises magnetosomes mostly comprised of magnetite and is surrounded by a bilayer membrane (Araujo et al., 2015). Toxicity testing of *in vitro* colon cancer models from HT-29 participants was not performed, and tumor necrosis was visible on both histologic slides and MRI (Mannucci et al., 2014). The biological characteristics of magnetic nanoparticles (MNPs) derived from magnetotactic bacteria were assessed using a multimodal method. Exposing magnetosomes (MN) to an alternating magnetic field (AMF) resulted in a noticeable increase in temperature. Furthermore, the temperature increase exhibited a solid linear relationship when tested in samples with increasing MN concentrations. Because of their iron concentration, chains of MNs are highly identifiable *in vivo* by MRI when injected directly into living tissue (Figure 4). This suggests that these nanoparticles could be used as a negative contrast agent or a magnetic tracer (Mannucci et al., 2014).

The expression of the MagA gene heightened the contrast of gene that encodes an iron transporter, which is positively regulated in the presence of low iron concentrations (Lohße et al., 2011). These magnetic properties have the potential to open up new clinical uses. T1 enhanced imaging revealed positive contrast features in AMB-1 strains cultivated in iron-deficient circumstances (Alphandéry, 2014). (Kraupner et al., 2017) recently compared the use of magnetosomes to trace magnetic particles in a new diagnostic imaging technology known as magnetic particle imaging to the gold standard commercial tracer Resovist. Magnetosomes resulted in a significant improvement in particle detection and, as a result, in the resolution of this approach, according to the data. According to several research, the MR-contrast obtained in the presence of MC-1 magnetotactic bacteria is mostly attributable to magnetic nanoparticles within the cell body. Even when non-magnetic MC-1 bacteria were more concentrated than magnetic MC-1 bacteria, their effect on the MR image was insignificant compared to the effect seen when the magnetosomes chain was present (Felfoul et al., 2010). The chain of magnetosomes is primarily responsible for the enhanced contrast *via* a resonant mechanism, where the magnetic resonance imaging signals are strongest within a specific frequency range. Many medical tasks, such as on-site delivery of MRI contrast agents, highly localised drug delivery for chemotherapy and chemoembolisation, thermal treatment of tumours at selected sites, and biosensing applications, could be enabled or improved by the use of special devices combining ferromagnetic materials and magnetotactic bacteria with being propelled in human blood vessels (Martel, 2006). This potential impact stems mostly from the fact that many remote areas within the human body are now inaccessible. The proposed carriers could potentially boost the efficacy of targeted medicines by navigating them *via* the human blood circulation system.

### Magnetosomes in Gene Therapy

It has been demonstrated that BMs could be used in gene therapy for gliomas. These tumors are particularly malignant. EGFR is one of the molecular variables contributing to gliomas’ invasiveness and poor prognosis in therapy (Xu et al., 2017). The initial build consisted of BMs coated with various nanocarriers, such as polyamidoamine dendrimers (PAMAM) and the Tat protein. PAMAM’s merits are its safety and low bulk, but Tat proteins have a greater potential to cross biological membrane barriers (Yan et al., 2015). Han et al. used siRNA-containing plasmids to create BM conjugates. EGFR expression in human glioblastoma u251-MG cells is suppressed by these plasmids (piRNAs) (Han et al., 2010). Gene therapy using BMs was also found to be successful in mice in these investigations. U251-MG xenografts transfected with Tat/BM/PAMAM-psiRNA-EGFR demonstrated a decrease in tumor volume, and immunohistopathological studies of protein expression *in situ* matched the *in vitro* results (Han et al., 2010). Another promising cancer-fighting method is gene therapy. It entails suppressing oncogenes and regulating transcription factors that are critical for tumor growth, among other things. This technique has one main flaw: introducing therapeutic components into target cells is difficult (Sun et al., 2019). Wang et al. presented a similar concept in using BMs for gene therapy. They developed BM-plasmid combinations that allowed cecropin B and apoptin (pVAX1–VA) to co-express. These proteins inhibited tumor growth by causing cell cycle arrest in the G2/M stages, death, and cell membrane disintegration (Wang et al., 2018). Hep G2 cell lines were transfected with a BM-associated pVAX1–VA plasmid, which efficiently restored clonal function. Apoptin and cecropin B were expressed at higher levels in the cells examined compared to control cells transfected with a lipofectamine-associated plasmid. Gene therapy using these proteins’ genes could be a promising treatment option for a variety of malignancies. Cecropin B has also been shown to boost apoptin activity (Birame et al., 2014).

The use of BMs to transport drug nanocarriers is linked to changes in their biological membranes, which change their properties in tissues, such as their stability and dispersibility. As a result, it is critical to pick molecules that do not affect the stability of BM-drug complexes. This is accomplished by keeping the zeta potential constant (Radeski et al., 2011). Furthermore, none of these chemicals should have cytotoxic properties. The clinical difficulties of utilizing nanoparticles to carry medications are discussed by (Park and Na, 2015; Pedziwiatr-Werbicka et al., 2021). Some of the most important considerations are biocompatibility, complex stability within blood serum and target tissues, half-life, the potential ability of nanoparticles to aggregate with platelets or aggregate within tissues, distribution of nanoparticles in tissues and organs, toxicity to normal tissues, interaction with the phagocytic system, and elimination pathways from the body.

### Magnetosomes in Hyperthermia Treatment

Cancer patients may benefit from hyperthermia treatment. It has no hazardous side effects, is less limiting than radiotherapy and chemotherapy, and can even be coupled to improve treatment efficacy (Johannsen et al., 2007). (Yukumi et al., 2009) were the first to describe the use of magnetic particles, hematite with a diameter of 20–100 nm, and magnetic particles to infuse heat into lymph nodes 60 years ago. The demise of lymphatic metastases followed this. Magnetosomes have been demonstrated to be effective in the treatment of cancers when utilized in hyperthermia. Magnetosomes that were individual, separated, and linked were used to treat a tumor containing the MDA-MB-231 breast cancer cell line (Alphandéry et al., 2011b). They were induced under the skin by mice. The tumor temperature reached 43°C when a 20 mT applied magnetic field at 198 kHz frequency for 20 min was used. Recent studies have revealed that magnetosomes coated with poly-L lysine are more stable, less pyrogenic, and more likely to create heat. This leads to a significant improvement in anticancer effects in mice with intracranial U87 Luc tumors. In this case, magnetosome hyperthermia treatment generated a temperature rise of 42°C in the tumors across 28 magnetic exposures (Alphandéry et al., 2017). SION (superparamagnetic iron dioxide nanoparticles) is a nanoparticle-based hyperthermia treatment that was created artificially. They are difficult to insert into tumors and are frequently ineffective. Furthermore, SION can cause adverse effects in people (Johannsen et al., 2007; Alphandéry et al., 2011b). (Mannucci et al., 2018) injected magnetosomes into U87MG cells in a glioblastoma model and subsequently exposed the animals to an alternating magnetic field for 2 weeks. Photothermal treatment was more effective than chemotherapy in suppressing PC3 cancer cells (human prostatectomy cell line). When genetically altered magnetosomes, modified by the fusing of an arginine-glycine-aspartic acid peptide coding gene to MamC were administered systemically and subjected to laser excitation, *in vivo* testing revealed significant tumor suppression (Espinosa et al., 2018). Coercivity (Hc) and the ratio of remanent to saturation magnetisation (Mr/Ms), which are proportional to the area of the hysteresis loops, often rise with increasing nanoparticle size. The quantity of heat generated by magnetosomes was calculated by measuring magnetosome losses per cycle, defined as magnetosome SAR (specific absorption rates) divided by the frequency of the applied magnetic field. Magnetosome losses each cycle increased with increasing magnetic field strength, from 0.1 to 0.2 J/kg (joules per kilogramme of iron contained in the heated magnetosomes) for a magnetic field strength of 6 mT to 0.5–1 J/kg for a magnetic field strength of 12 mT (Hergt et al., 2008; Sun et al., 2009; Timko et al., 2012). However, it was discovered in some investigations that when a magnetic nanoparticle assembly is injected into a tumour, uncontrolled agglomeration of nanoparticles occurs, resulting in the creation of dense clusters of nanoparticles of varied geometric forms (Gudoshnikov et al., 2012; Etheridge et al., 2014; Sanz et al., 2016). As a result of the high magnetic dipole interaction between the cluster’s nanoparticles, the SAR value of the assembly is dramatically reduced.

As detailed in this article, Magnetosomes can bind to proteins that detect specific cells or organs inside the organism. Magnetosomes can thus be utilized to induce hyperthermia or as delivery methods. As a result, treatments that target malignant or damaged tissues could be targeted (Lee et al., 2015; Yoshino et al., 2018).

### Magnetotactic Bacteria in Novel Technologies

MTB cells are still being studied in a variety of novel and sometimes odd methods. For example, (Smit et al., 2018) proposed utilizing MTB cells to create low voltage power based on Faraday’s law of electromagnetic induction. (Pierce et al., 2017) recently improved the hydrodynamics of motile MTB cells. This was accomplished by the use of magnetic fields to control their motility. This highlighted MTB cells’ utility in the creation of functional micro-robotic technologies. (Blondeau et al., 2018) revealed that manipulating magnetosome chains in silica-encapsulated MTB cell cells did not affect cell viability, raising the prospect of functional devices in the future.

### Magnetosomes in Detection Assays

Magnetosomes have been successfully used in protein detection methods (Ceyhan et al., 2006). Using biotin groups linked to the magnetosome membrane, the protein streptavidin was bound to magnetosomes. These biotin-binding semisynthetic composite particles could be used to connect a wide range of functional biomolecules, including biotinylated DNA oligonucleotides and biotinylated antibodies (Ceyhan et al., 2006). In this method, antibody-functionalized magnetosomes were used to fix HBsAg (hepatitis B antigen) in human serum and magnify the signal supplied by the detecting complex *via* magnetic concentration (Wacker et al., 2007). Magneto Immuno PCR (M-IPCR) detected HBsAg approximately 100 times more sensitively than magneto-ELISA, which used synthetic nanoparticles to increase antigen detection in ELISA and was run in tandem with M-IPCR for comparison (Liu et al., 2010). This technique has the potential to be beneficial in immunological diagnostics and proteome research. An automatable, highly sensitive immuno-PCR (M-IPCR) modification was established using antibody-functionalized magnetosomes in a surface-independent immunoassay (Wacker et al., 2007). Antibodies coupled to magnetosomes or magnetosome crystals have been produced and have shown useful in various immunoassays involving allergen detection, cancer cell detection, and immunoglobulin quantification (Leão et al., 2020). The use of magnetic fields enabled measurements of the change in light scattering caused by cell alignment in a magnetic field or the change in absorbance caused by bacteria swimming across the light beam. MSP was found to be a powerful technique for determining bacterial magnetism and analysing the alignment and swimming of magnetotactic bacteria in magnetic fields (Lefèvre et al., 2009). Furthermore, we were able to characterise south-seeking derivatives and non-magnetosome-bearing strains derived from north-seeking MO-1 cells using this test. In another study, modified BMPs such as F-BMP-FA were found to be 90% more effective than commercial immunomagnetic beads, with a detection sensitivity of 5 CFU/mL. F-BMP-FA also formed a compound with Abs from crude mouse ascites. The lowest gentamicin sulphate detection line for BMP-A-Ab was 0.01 ng/ml, which is 400-fold lower than the detection line for double Ab sandwich ELISA, and the recovery rate for gentamicin sulphate for BMP-A-Ab was 93.2 percent (Xu et al., 2019).

### Magnetosomes Involved in Enzyme Immobilization

The protein display technology of magnetosomes can be employed to express catalytic units. As a result, they are excellent candidates for supporting immobilized enzymes. Magnetic nanoparticles including magnetosomes have been widely used as support materials for enzyme immobilization due to their ease of recovery (Johnson et al., 2011). Ginet and co-workers discovered an organophosphohydrolase from *Flavobacterium* sp. This protein complex was coupled with MamC to decrease paraoxon which is a hazardous but frequently used compound (Ginet et al., 2011). The rate of paraoxon breakdown in this protein complex was comparable to that of purified organophosphohydrolase. Honda and his colleagues studied the feasibility of producing biofuels utilizing magnetosome-enzyme complexes (Honda et al., 2015). Magnetosomes were used to generate a multi-enzyme complex, and peptides were genetically linked to MamC through a peptide bridge. These peptides were then employed to bind beta-oxidase or endoglucanase enzymes to the complex. The cellulose-degrading activity of this complex was substantially higher than that of the non-immobilized enzymes assessed individually. After five cycles of use, 70% of the cellulose-degrading activity of the multi-enzymatic compound was still detectable.

Because of their unique magnetic property, MNPs stand out from other types of nanoparticles. MNPs have two major drawbacks: burst drug release and limited stability. Surface ligands are linked to MNPs to solve this problem, which improves their stability and solubility in biological contexts while also reducing negative effects (Yang et al., 2018). Chemotherapy, radiation, and medical procedures are the three therapeutically available treatments for tumor control at the moment. The main disadvantages of these treatments are the unspecific side effects. Hyperthermia, in which the temperature of a local region or the entire body is raised to 40–45°C using radiation, can be used to achieve this. The second procedure, thermo-ablation, destroys tissues by applying temperatures exceeding 45°C to the afflicted area (Cardoso et al., 2018). Their magnetic properties are useful not only for magnetic separation and magnetic resonance imaging, but also for tissue engineering, gene transfection, magnetic memory devices, and magnetic ink, among other things. MNPs can be used for drug targeting and cell sorting, among other things. MNP-mediated hyperthermia has been utilized to successfully treat mouse cancers in animal models (Arruebo et al., 2007). Human epidermal growth factor receptor 2 (HER2) antibody coupled with paclitaxel (PTX) or rapamycin loaded glycerol mono-oleate-coated magnetic nanoparticles (GMO-MNPs) exhibited 24 times more potent anticancer activity than the free drug (Dilnawaz et al., 2010). Nanotoxicity is a real possibility that should be taken seriously; yet, research in this interesting field continues.

MNPs have a significant surface-to-volume ratio, which means they have a lot of chemically active sites for biomolecule conjugation which allows for a longer period of circulation, target-specific binding, and drug administration.

## Conclusion

This review discussed the therapeutic applications of magnetotactic bacteria and their by-products, bacterial magnetosomes (BMs). The rising incidence of cancer is one of the issues facing modern medicine, especially since standard treatments are sometimes inefficient or impossible to execute. Designing effective distribution systems for therapeutic chemicals is one of the novel research directions in boosting the effectiveness of cancer treatment. Magnetotactic bacteria and their special organelles, known as magnetosomes, contain ferromagnetic crystals, offer much potential in this field. MTBs and BMs, which are biocompatible, can be employed as natural nanocarriers to deliver chemotherapy to the cancer cell’s target site with excellent precision. Classic anticancer medications, gene therapy, drug delivery, detection assays, magnetic resonance imaging, and hyperthermia treatment can all be changed and coupled with MTBs and BMs. Some of the advantages of MTB and their magnetosomes include targeted drug delivery, reduction of drug toxicity, tissue specificity, reutilization of capture complex; High specificity separation. These magnetosomes also be used as therapeutic tool by hyperthermia, drug delivery, high affinity to target cells, high detection sensitivity, The whole MTB has its advantages in reutilization of nanobiocatalyst, immobilization of multiple catalysts, Magnetic crystal doping possible, recovery of removed minerals and green technology. These also show less significant side-effects than chemotherapy and radiotherapy. The limitations of these include difficulty in cloning and expression, alteration of cell viability after capture, and some possible loss of activity due to immobilization.

Furthermore, because of their ferromagnetic features, these microbes can be manipulated inside a magnetic field. This opens up many opportunities to create constructs, which is part of the concept of targeted cancer therapy. While bio-synthesized IONP has been described as biocompatible and capable of inducing cytotoxicity in tumour cells under certain conditions, a detailed assessment of the anti-tumour activity that such NP could trigger has only been done for magnetosomes, with results demonstrating that it was possible to completely eradicate certain types of tumours in mice or subcutaneously by administering the NP. This treatment was effective while also having no obvious side effects, probably because of the mild heating temperatures reached during treatment and magnetosome biocompatibility.
